# The association between BMI and mortality using early adulthood BMI as an instrumental variable for midlife BMI

**DOI:** 10.1038/s41598-018-29089-z

**Published:** 2018-07-31

**Authors:** Marte K. R. Kjøllesdal, George Davey Smith, Inger Ariansen, Jonas Minet Kinge, Eirik Degerud, Øyvind Næss

**Affiliations:** 1 Institute of Health and Society, University of Oslo, Oslo, Norway; 2 MRC Integrative Epidemiology Unit (IEU), at the University of Bristol, Bristol, UK; 3 Domain for Mental and Physical Health, Norwegian Institute of Public Health, Oslo, Norway

## Abstract

The article aims to describe the association between midlife body mass index (BMI) and cardiovascular disease (CVD)- and all-cause mortality, and to use early adulthood BMI as an instrumental variable for midlife BMI, in order to obtain an estimate less distorted by midlife confounders and reverse causality. Data from Norwegian health surveys (1974–2003) (midlife BMI, smoking, blood pressure, total cholesterol, heart rate), Military Conscription Records, National Tuberculosis Screenings (early adulthood BMI), National Educational Registry and Cause of Death Registry were linked. Participants with data on BMI in early adulthood and midlife were included (n = 148.886). Hazard Ratio (HR) for CVD mortality was higher in men with midlife obesity relative to normal weight (HR = 1.46(95% CI 1.25, 1.70). For all-cause mortality, HR was higher in those with obesity or underweight in midlife relative to normal weight (Men:HR = 1.19(95% CI 1.09, 1.29), HR = 2.49(95% CI 1.81, 3.43) Women:HR = 1.33(95% CI 1.13, 1.56), HR = 1.61(95% CI 1.22, 2.13)). In instrumental variable analyses, increased BMI became more strongly associated with CVD and all-cause mortality, and the increased risk of all-cause mortality among the underweight attenuated.

## Introduction

A number of studies have reported a J-shaped or U-shaped association between Body Mass Index (BMI) and mortality from all-causes and several specific causes, including cardiovascular diseases (CVD)^1–5^. Most of the evidence on the risk associated with different levels of BMI comes from observational studies where measurements of BMI have been conducted in midlife. This may cause biased estimates on the role of BMI because BMI may become increasingly related to a number of confounders through the life course. Possible confounders could be health related behaviors and socioeconomic position (SEP)^6,7^. A probable important contributor to excess deaths in the lowest weight groups is illness, leading to both unintentional weight loss and increased mortality^8^. Further, weight trajectories over time could influence mortality risk. For example, people with previous obesity who have lost weight may upwardly bias mortality risk amongst those of normal weight^8,9^.

In order to circumvent these sources of bias, some have excluded the first years of follow-up, as ill people have a high risk of dying during these years^10^. However, illness causing low BMI can last for a longer period than the time excluded. This approach could also exclude deaths from illness caused by obesity, and thus attenuate the risk associated with high BMI^8^. Others adjust for possible confounders at baseline or exclude sub-groups, such as smokers. Assessment of confounders may, however be incomplete or inaccurate and adjusted analyses may retain considerable residual confounding^11^.

Instrumental variable analysis is an approach that may ideally handle misclassification, confounding and reverse causality, and thus provide an alternative estimate of the association between BMI and mortality. An instrumental variable is ideally associated with the risk factor of interest, and only through that, to the outcome under study. It should not be related to confounding variables^12^. BMI is influenced by many factors acting over the life course, and also subject to genetic variation, such as the FTO-gene^13^. Studies based on the principles of Mendelian randomization^14^ have used genetic variants as instruments for BMI and thus strengthened evidence for a causal effect of BMI on CVD^15–17^. Others have used offspring BMI and offspring blood pressure as instruments for own BMI and blood pressure to investigate its association with mortality^18,19^ and employment disability^20^.

Here we use an alternative instrument for own midlife BMI, namely own BMI in early adulthood, to estimate the association between BMI and all-cause mortality and CVD mortality. Both offspring weight and early adulthood BMI may be related to confounding factors. However, both mitigate against the problem of reverse causality by illness developing in adulthood, which may be the major problem in studies of BMI and mortality. An advantage of early adulthood BMI is that it is often more available than offspring weight. Further, it does not exclude those who have no children, who may have different characteristics to those with children. The assumptions that should be in place for a variable to be used as an instrument are listed in Table 1.

**Table 1:** Adherence to instrumental variable assumptions.

Assumptions	How assumptions are met in this study
The instrumental variable is associated with the risk factor of interest	In a number of studies, BMI in early life is positively associated with BMI later in life^37^. In our study, the F-test suggested the instrument was strong.
The instrumental variable is not influenced by the outcome	BMI in early adulthood is clearly not influenced by CVD mortality later in life, even though early process of disease stages could influence BMI.
The instrumental variable is not associated with confounding factors	Possible confounders of the association between BMI in midlife and subsequent CVD mortality are health related lifestyle, illness and socioeconomic position (SEP). Lifestyle and SEP in midlife cannot affect BMI in early adulthood directly. However, lifestyle and SEP in early life may influence lifestyle and SEP later in life. Thus, this approach is not sufficient to deal with all possible confounders tracking over the life course. CVD risk factors were found to be more strongly associated with BMI in midlife than in early adulthood, suggesting that associations with possible confounders are stronger in midlife than earlier. The instrument is not associated with disease in midlife influencing both BMI and mortality.
There is no pathway from the instrumental variable to the outcome, except through the risk factor.	We cannot demonstrate that there is no direct link between BMI in early adulthood and later CVD mortality. However, recent reviews of studies on the association between BMI in childhood and adolescence and later risk of CVD have found that there is little evidence for childhood obesity being an independent risk factor for adult cardiovascular risk, when adult BMI is accounted for^38,39^. Thus, the association between BMI in early adulthood and later CVD mortality appears to mainly reflect its association with BMI later in life.

In a large sample of Norwegian women and men with objective measurements of BMI in early adulthood and in midlife, as well as measurements of midlife CVD risk factors, we first aim to describe the association between midlife and early adulthood BMI and cardiovascular and all-cause mortality. Our second aim is to give an estimate of the association between midlife BMI and all-cause and cardiovascular mortality, using BMI in early adulthood as an instrumental variable for BMI in midlife.

## Methods

### Variables

We included data from participants in national and regional Norwegian cardiovascular health surveys. In the Counties Study (1985–88), all men and women aged 35–49 years living in three different counties in Norway were invited to a survey^21^. In the Age 40 Program, inhabitants aged 40–44 years in all Norwegian counties, except for Oslo, were invited (1985–99)^22^. The Cohort Norway (CONOR) (1994–2003) is based on data from health surveys from all over Norway, with participants aged 20–103 years^23^. The attendance rate of the three surveys was 86%, 70% and 58% respectively^23–25^.

The surveys assessed CVD risk factors. Self-reported smoking status (“daily smoker” “not daily smoker”) and current treatment of hypertension was recorded. Blood pressure (BP, mmHg) was measured by automatic oscillometric devices, and the average of the last two available measures defined BP. Resting heart rate was recorded (beats/minute). Non-fasting serum total cholesterol (TC, mmol/L) was initially measured by non-enzymatic, and later enzymatic, method. Non-enzymatic values were converted by a correction factor. BMI was calculated from objective measurements of weight and height (kilogram/meter^2^).

Men enrolled for military service, usually between 18 and 20 years of age, are obliged to complete several conscript examinations, including objective measurements of height and weight. About 90% of Norwegian men participate in the conscript examinations. Those not undergoing this appraisal include those who are physically and mentally disabled, have a criminal record or being abroad at the normal conscript age^26^. During 1963–1975, there was compulsory screening for tuberculosis in Norway, with population coverage for persons ≥15 years, which included objective measurements of height and weight. Health personnel travelled through the country carrying out these assesments^27^. BMI in early adulthood was calculated from weight and height at conscription, or if not available, from data from tuberculosis screenings for those aged 18–20 years at the time of examination. As the conscript data was almost exclusively men, our final sample consisted of about four times as many men as women. BMI both in early adulthood and midlife was categorized into weight groups; “underweight <18.5 kg/m^2^”, “normal weight 18.5 to <25 kg/m^2^”, “overweight 25 to <30 kg/m^2^” and obesity ≥30 kg/m^2^”.

Education was registered in the National Educational Registry and reported in National Population and Housing Censuses every 10^th^ year from 1970 to 2011. A person’s highest attained educational level was classified as “≤9 years”, “10–11 years”, “12 years”, “13–16 years” and “>16 years”.

Data on underlying causes of death from CVD were obtained from the Norwegian Cause of Death Registry (ICD 8: 390–444.1, 444.3–458, 782.4, ICD-9: 390–459, ICD-10: I00-I99). Participants were followed up for all-cause and CVD mortality from their participation in a health survey in midlife until December 31^st^ 2014.

### Sample

**Figure 1:**
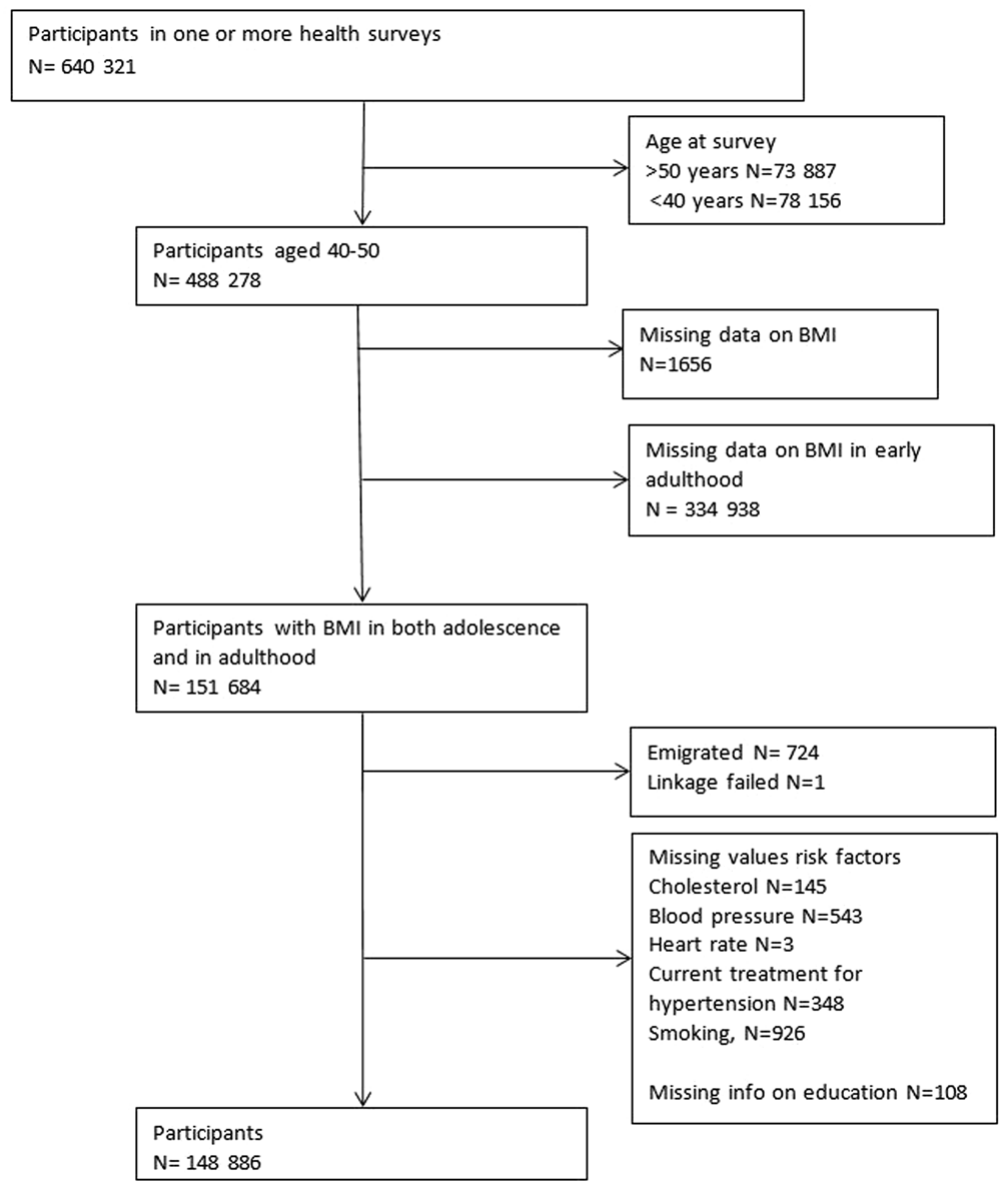
Flow chart.

Data from health surveys, conscript data, tuberculosis screenings, the National Educational Registry and the Cause of Death Registry were linked using the unique national personal identity number. Participants from the health surveys turning 40–50 years at the year of their survey were selected. For those attending more than one health survey, data from their first survey was used. Only persons with data on BMI both in early adulthood and midlife were included. Those with missing data on education or any of risk factors were excluded (1.37%). The total number of participants was 148 886 (Fig. 1).

### Statistical analyses

Cox proportional hazards regression models were used to calculate hazard ratio (HR) and 95% confidence intervals (CIs) for all-cause and cardiovascular mortality for each weight group (underweight, overweight and obesity), with normal weight as the reference, both in early adulthood and midlife. The models used age as underlying time, and robust variance estimation. Time (years of age) at risk was counted from when a participant entered the study. Individuals who did not die during follow up were censored at their age at the end of follow-up. Mean follow-up time was 19 years. Analyses were adjusted for education and CVD risk factors (smoking, serum total cholesterol, systolic and diastolic blood pressure, current treatment for hypertension, heart rate and height). Sensitivity analyses were carried out, with adjustment for index health survey and time of survey. Sensitivity analysis were also carried out on percentiles of BMI in midlife and early adulthood, looking at the highest and the lowest BMI groups, captured as the 10^th^ and 90^th^ percentile, as well as the 50–90^th^ percentile compared to the 10–50^th^ percentile. The association between cardiovascular death and BMI was explored using Poisson regression with robust variance estimation, and incidence rate ratios (IRR) were calculated.

BMI in midlife was instrumented by early adulthood BMI using an instrumental variable Poisson model based on a two-step GMM estimator (IV-poisson). Poissons regressions were adjusted for education. Prior research suggests that the relationship between body weight and health status is nonlinear^2^. To accommodate nonlinearities in the relationship between mortality and BMI we estimated a second set of Poisson and IV-Poisson models in which the endogenous regressors were the participant’s BMI and BMI squared. As we had two endogenous regressors (BMI and BMI squared) we needed a second instrument and created a squared term in our IV regressions by predicting the endogenous variable in our first stage regression, then used the square of the predicted term as an IV following (in two-stage least squares framework) Wooldridge^28^. Margin plots with predicted number of events were made for CVD and all-cause mortality based on Poisson and IV-Poisson regressions. To assess the extent of bias from confounders, we compared the covariate balance by levels of BMI and instrument, using Ordinary Least Square regression and instrumental variable analyses (Suppl. Fig. A), showing smaller bias when using the instrumental variable approach for all confounders except education (Hausman test)^29^. The F-test of the instrument, based on a linear first stage model, was 36803, considerably exceeding conventional criteria.

To demonstrate the practical implications of using the instrumental variable approach, we envisaged a scenario where all participants increased their BMI by 2 units. The predicted number of events over the BMI scale was used to predict number of total deaths in our sample, both with measured BMI and with increased BMI, using the conventional and the instrumental variable approach.

STATA 14 was used for data analyses.

The study was approved by the Norwegian Regional Committees for Medical and Health Research Ethics (REK) (2012/827). Written informed consent was obtained from participants in the Age 40 Program and CONOR. Participants in the Counties studies gave written permission for their results to be sent to their physician. Permission to be absolved from this professional secrecy has been granted and concession to handle this personal health information has been given by the authorities. The health studies have been conducted in full accordance with the World Medical Association Declaration of Helsinki.This study is part of a research project at the Norwegian Institute of Public Health (NIPH). The linked data used in the study can be made available on a remote access platform to researchers who become project members. The project is responsible for obtaining for new members the necessary approvals from the Regional Ethics Committee South-East as well as to ensure that the new member sign a confidentiality agreement with Statistics Norway. It is also possible to apply for data from NIPH and Statistics Norway.

## Results

The majority of participants were normal weight in early adulthood, however, in midlife more than half of men and about one third of women had overweight or obesity (Table 2). The number of CVD deaths during follow up was 1473 among men and 199 among women, and the total number of deaths was 5829 and 1709, respectively. The risk factors were more strongly associated with BMI in midlife than in early adulthood (Table 3). A stronger association between covariates and BMI in midlife than in early adulthood was seen in all weight categories, except for smoking among those with overweight and obesity, and diastolic BP among those with overweight (Suppl. Table A).

**Table 2:** Characteristics of the sample, men and women.

	Men N = 116 777	Women N = 32 109
Birth year, min max	1943–1963	1943–1962
Examination year, min max	1985–2003	1985–2003
Age by examination, mean (SD)	41.4 (1.3)	41.7 (1.4)
BMI in midlife (40–50 years), mean (SD)	26.0 (3.3)	24.3 (3.9)
BMI in early adulthood (18–20 years), mean (SD)	21.6 (2.3)	21.8 (2.7)
Weight category, early adulthood, n (%)		
Underweight	6576 (6)	2253 (7)
Normal weight	102 017 (87)	26 342 (82)
Overweight	7496 (6)	3192 (10)
Obesity	694 (1)	322 (1)
Weight category, midlife, n (%)		
Underweight	269 (0)	556 (2)
Normal weight	47 984 (41)	20 621 (64)
Overweight	56 082 (48)	8314 (26)
Obesity	12 442 (11)	2618 (8)
Education, n (%)		
≤9 years	15 217 (13)	6145 (19)
10–11 years	34 873 (30)	14 593 (45)
12 years	36 381 (31)	4632 (14)
13–15 years	21 484 (18)	6132 (19)
≥16 years	8822 (8)	607 (2)
CVD mortality, n (%)	1473 (1)	199 (0)
All-cause mortality, n (%)	5829 (5)	1709 (5)
Age at death, mean (SD), min max	54.1 (6.0) 39–68	56.1 (6.3) 40–68
Age at CVD death, mean (SD), min max	54.0 (6.0) 39–68	56.5 (6.3) 41–68
**Midlife risk factors**		
Blood pressure (mmHg)		
Systolic, mean (SD)	135 (13)	127 (14)
Diastolic, mean (SD)	80 (10)	78 (10)
Heart rate (beats/min), mean (SD)	72 (12)	78 (13)
Cholesterol (mmol/L), mean (SD)	5.8 (1.1)	5.5 (1.0)
Height (cm), mean (SD)	179.3 (6.4)	165.8 (5.7)
Daily smoking, n (%)	44 459 (38)	13 074 (41)

BMI: Body Mass Index CVD: Cardiovascular Disease.

**Table 3:** Crude associations between risk factors for cardiovascular disease and Body Mass Index (BMI) measured in midlife and in early adulthood.

	BMI (kg/m^2^)	
	Early adulthood (18–20 years)	Midlife (40–50 years)
	β (95% CI)	
Serum total cholesterol (mmol/L)	0.03 (0.02, 0.03)	0.06 (0.06, 0.07)
Systolic Blood Pressure (mmHg)	0.57 (0.54, 0.60)	1.13 (1.11, 1.15)
Diastolic Blood Pressure (mmHg)	0.39 (0.37, 0.41)	0.72 (0.71, 0.74)
Heart Rate (beats/min)	0.07 (0.04, 0.09)	0.20 (0.19, 0.22)
BMI, early adulthood (kg/m^2^)	0.38 (0.38, 0.39)	
	Odds Ratio (95% CI)	
Smoking, ref: no	1.01 (1.01, 1.02)	0.94 (0.94, 0.94)
Current treatment for hypertension, ref: no	1.16 (1.15, 1.17)	1.19 (1.18, 1.20)

Total cholesterol, systolic and diastolic blood pressure, heart rate, BMI early adulthood: linear regressions. Smoking and current treatment for hypertension: logistic regressions.

### Weight categories and mortality

Table 4 shows the association between CVD- and all-cause mortality and BMI in early adulthood and midlife. Among men, those with overweight (HR = 1.37 (95%CI 1.16, 1.62)) or obesity (HR = 2.70 (95% CI 1.88, 3.78)) in early adulthood, as well as those who with underweight (HR = 2.38 (95% CI 1.12, 5.05)) or obesity in midlife (HR = 1.46 (95% CI 1.25, 1.70)), had higher HR of CVD mortality than normal weight men, after adjustment for education and CVD risk factors. These differences were not seen among women.

**Table 4:** Hazard ratios (HR) for all-cause and cardiovascular (CVD) mortality according to weight categories from Cox regressions.

CVD mortality	Model 1	Model 2	HR (95% CI)	Model 1	Model 2	Model 3
			Model 3			
	Men			Women		
Midlife						
Underweight	2.54 (1.21, 5.35)	2.24 (1.06, 4.72)	2.38 (1.12, 5.05)	2.45 (1.45, 5.25)	2.23 (1.04, 4.80)	2.09 (0.96, 4.51)
Normal weight	1	1	1	1	1	1
Overweight	1.10 (0.98, 1.24)	1.05 (0.94, 1.19)	0.92 (0.82, 1.04)	1.19 (0.86, 1.66)	1.11 (0.80, 1.54)	0.88 (0.63, 1.24)
Obesity	2.40 (2.08, 2.77)	2.18 (1.88, 2.51)	1.46 (1.25, 1.70)	2.31 (1.55, 3.44)	2.00 (1.34, 2.97)	1.21 (0.78, 1.89)
Early adulthood						
Underweight	1.16 (0.93, 1.45)	1.12 (0.89, 1.39)	1.17 (0.94, 1.46)	1.11 (0.64, 1.92)	1.02 (0.59, 1.77)	1.07 (0.62, 1.86)
Normal weight	1	1	1	1	1	1
Overweight	1.92 (1.63, 2.36)	1.79 (1.52, 2.11)	1.37 (1.16, 1.62)	1.32 (0.86, 2.01)	1.20 (0.79, 1.84)	0.95 (0.62, 1.46)
Obesity	4.41 (3.12, 6.23)	3.86 (2.73, 5.45)	2.70 (1.88, 3.78)	3.79 (1.78, 1.10)	3.06 (1.44, 6.50)	1.81 (0.82, 3.97)
**All-cause**	**Men**			**Women**		
Midlife						
Underweight	3.09 (2.25, 4.25)	2.77 (2.01, 3.81)	2.49 (1.81, 3.43)	1.93 (1.46, 2.55)	1.85 (1.40, 2.44)	1.61 (1.22, 2.13)
Normal weight	1	1	1	1	1	1
Overweight	0.95 (0.90, 1.01)	0.93 (0.87, 0.98)	0.91 (0.86, 0.96)	1.00 (0.90, 1.13)	0.97 (0.86, 1.08)	0.94 (0.84, 1.06)
Obesity	1.49 (1.38, 1.61)	1.37 (0.27, 1.49)	1.19 (1.09, 1.29)	1.56 (1.34, 1.82)	1.45 (1.24, 1.69)	1.33 (1.13, 1.56)
Early adulthood						
Underweight	1.14 (1.02, 1.27)	1.11 (0.99, 1.23)	1.10 (0.98, 1.22)	0.10 (0.91, 1.32)	1.05 (0.87, 1.27)	1.03 (0.86, 1.24)
Normal weight	1	1	1	1	1	1
Overweight	1.45 (1.33, 1.59)	1.37 (1.25, 1.50)	1.21 (1.11, 1.33)	1.25 (1.08, 1.45)	1.19 (1.03, 1.38)	1.10 (0.94, 1.27)
Obesity	2.47 (0.97, 3.09)	2.20 (1.76, 2.75)	1.82 (1.45, 2.27)	2.26 (1.62, 3.14)	2.01 (1.45, 2.81)	1.71 (1.22, 2.38)

Model 1: crude, Model 2: adjusted for education, Model 3: adjusted for education and risk factors. P-value for difference in observational estimates and IV estimates <0.01 for all-cause mortality among women and men, and cardiovascular mortality among men.

In midlife, all-cause mortality risk was higher among those with underweight and obesity than among normal weight men (HR = 2.49 (95% CI 1.81, 3.43), HR = 1.19 (95% CI 1.09, 1.29), respectively) and normal weight women (HR = 1.61 (95% CI 1.22, 2.13), HR = 1.33 (95% CI 1.13, 1.56), respectively), when adjusted for education and CVD risk factors. Men with overweight had a lower mortality risk compared to normal weight men (HR = 0.91 (95% CI 0.86, 0.96)). In early adulthood, all-cause mortality was also higher among individuals with obesity in comparison to normal weight individuals among both men (HR = 1.82 (95% CI 1.45, 2.27)) and women (HR = 1.71 (95% CI 1.22, 2.38)) and among men with overweight (HR = 1.21 (95% CI 1.11, 1.33)). We experimented with one extra weight group, namely BMI > = 35 kg/m2. The results were in essence the same, however more pronounced in the highest weight group than among those with BMI 30–34.9 kg/m2. Due to few participants with a BMI > = 35 in early adulthood, we analyzed obesity as one group.

To account for different distribution of early adulthood and midlife BMI, we ran sensitivity analyses on percentiles of observed BMI in midlife and early adulthood, examining the highest and the lowest BMI groups, captured as the 10^th^ and 90^th^ percentile, as well as the 50–90^th^ percentile compared to the 10–50^th^ (Suppl. Table B). The results remained essentially the same. However, the tendency that HR estimates for obesity compared to normal weight were higher in early adulthood than in midlife were not replicated when comparing the 90^th^ percentile to the 10–50^th^ percentile. Further, an association between underweight in midlife among men and obesity in early adulthood among women and all-cause mortality was not replicated comparing the 10^th^ and the 90^th^ percentile to the 10–50^th^ percentile.

### BMI and mortality

Higher BMI in early adulthood and in midlife was associated with an increased CVD mortality among both men and women (Table 5).

**Table 5:** Associations between Body Mass Index (BMI) and cardiovascular mortality and all-cause mortality, Poisson regression coefficients and incidence rate ratio (IRR) from Poisson regressions (additive residuals).

Cardiovascular mortality	Coefficient (95% CI)	IRR	Coefficient (95% CI)	IRR
	Men		Women	
Early adulthood BMI	0.09 (0.07, 0.11)	1.10	0.06 (0.01, 0.11)	1.06
Midlife BMI	0.07 (0.05, 0.08)	1.07	0.05 (0.01, 0.08)	1.05
Early adulthood BMI as instrumental variable for BMI	0.10 (0.08, 0.13)	1.09	0.07 (0.02, 0.12)	1.04
**All-cause mortality**	**Men**		**Women**	
Early adulthood BMI	0.05 (0.04, 0.06)	1.05	0.03 (0.01, 0.04)	1.03
Midlife BMI	0.01 (0.01, 0.02)	1.01	0.01 (−0.00, 0.02)	1.01
Early adulthood BMI as instrumental variable for BMI	0.06 (0.04, 0.07)	1.05	0.03 (0.01, 0.05)	1.03

Instrumental variable analyses, using early adulthood BMI as an instrument for midlife BMI, ivpoisson regressions (multiplicative residuals) in STATA. All associations adjusted for education.

Among both women and men, higher BMI in early adulthood was associated with higher all-cause mortality (Table 5). Midlife BMI was associated with higher all-cause mortality only in men. Using the instrumental variable approach, midlife BMI was also associated with higher all-cause mortality among women. The estimates from the instrumental variable analyses were higher than the observational estimates for all-cause mortality among women and men, and for cardiovascular mortality among men.

**Figure 2:**
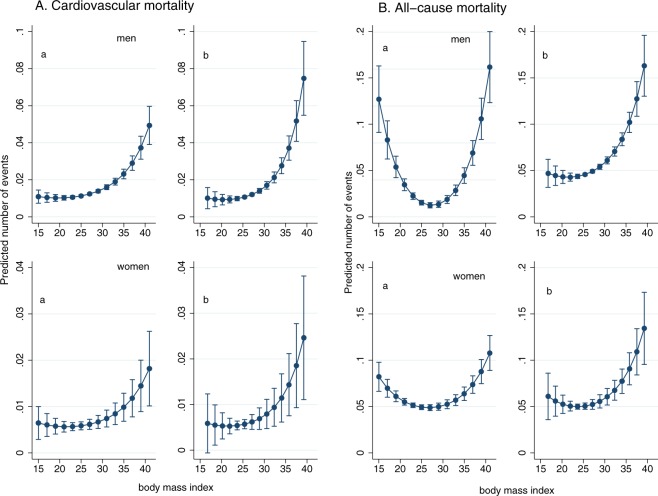
(**A**) Predictive margins for cardiovascular mortality by (a) body mass index (BMI) in midlife and (b) BMI in midlife instrumented by BMI in early adulthood, among men and women. Adjusted for education. (**B**) Predictive margins for all-cause mortality by (a) body mass index (BMI) in midlife and (b) BMI in midlife instrumented by BMI in early adulthood, among men and women. Adjusted for education.

Figure 2 depicts the relationships between BMI in midlife and CVD and all-cause mortality when assessed using the conventional approach and the instrumental variable approach. For CVD, the associations were J-shaped among the women and linear among the men. The association between midlife BMI and CVD mortality was stronger with the instrumental variable approach than with the conventional approach.

For all-cause mortality, a U-shaped association with midlife BMI was seen using the conventional approach. With the instrumental variable approach, the association between BMI and all-cause mortality was slightly stronger than in conventional analyses, and mortality in lower weight groups did not differ considerably from normal weight subjects. The lowest mortality risk was at lower BMI levels using the instrumental variable approach than the conventional approach. It was in the upper end of the normal weight range among women and in the middle of the normal weight range among men.

In sensitivity analyses, adjusted for index survey, and year of survey, estimates were not markedly changed (results not shown). Finally, we reran our IV-models using linear two-stage least squares and this did not change the conclusions.

**Figure 3:**
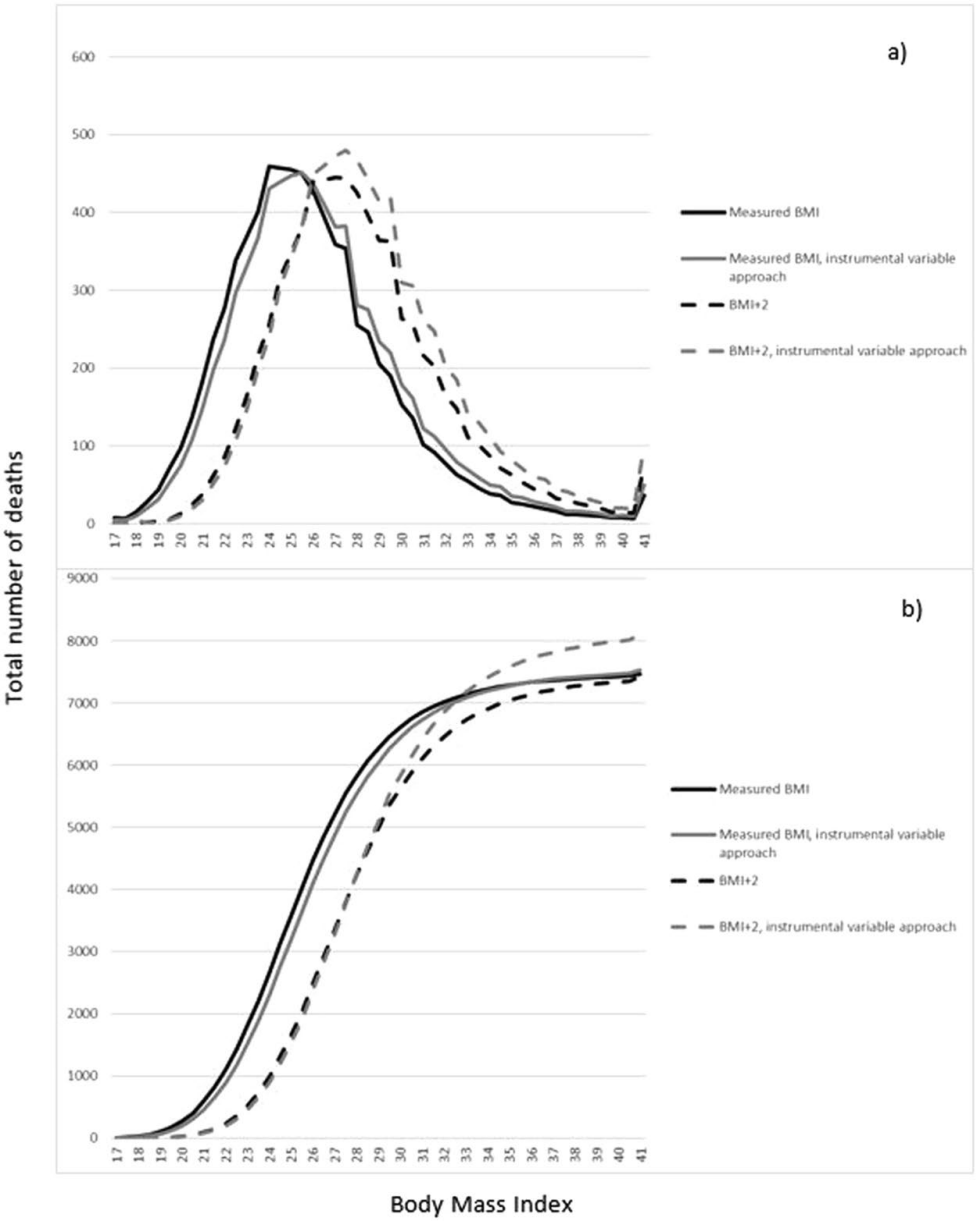
Number of total deaths over BMI units, measured BMI and in a scenario were all participants have gained weight according to 2 BMI units, using conventional and instrumental variable approach. Based on predicted number of events. (**a**) predicted number of deaths at each BMI unit and (**b**) cumulative predicted number of events.

To evaluate the impact of using either the conventional approach or the instrumental variable approach, we imposed a scenario in which the midlife BMI in the study population increased with two units, and compared the number of predicted deaths at different levels of BMI with the two approaches (Fig. 3). The instrumental variable approach predicted that more subjects would die from overweight and obesity than the conventional approach.

## Discussion

This study suggests that the effect of BMI on all-cause mortality, and especially cardiovascular mortality, might have been previously underestimated. Stronger estimates in instrumental variable analyses may be due to circumvention of midlife confounders, and to the included aspect of exposure over time. The results suggest that confounding caused by illness-induced low weight was reduced by using early adulthood BMI as an instrument for midlife BMI.

The study had a large sample of men and women from all areas in Norway, with measured data on BMI in early adulthood and midlife. The majority of previous studies on weight at different stages of the life course have been based on self-report of weight, at least at one time point^30–33^. Use of register data largely eliminates problems related to loss to follow-up. Moreover, the health surveys with data on risk factors had a reasonable response rate. However, many subjects did not reach a high age where CVD mortality is more probable during follow-up and our results are essentially confined to premature CVD- and overall mortality. Associations between BMI and mortality changes with age^3^. Therefore, our estimated associations would probably change if we followed the participants into older ages.

The shape of the naïve observational associations between BMI and CVD mortality^1^. and between BMI and all-cause mortality^2,3,5^, are in line with previous studies. The increased all-cause mortality with increasing weight is largely attributable to CVD^1^. Blood pressure, smoking, and to some extent LDL cholesterol, have been found to explain a large share of the association between BMI and CVD in adults^34,35^.

Smoking could be one explanation for higher mortality in low weight groups. Removing smokers from analyses has attenuated the increased risk in low weight groups in previous studies^2^. In our study, adjustment for CVD risk factors, including smoking, did not substantially change the increased risk of all-cause mortality among underweight.

When adjusted for education, the increased risk of CVD mortality among women with obesity was no longer observed. Both obesity and CVD mortality are more common in lower SEP groups, so education is a plausible confounder in this association^7^. A confounding effect of risk factors, as well as of SEP, on the association between BMI and mortality is probably related to health behavior during the life course. The effect of such possible confounders, both adjusted for (smoking) and not adjusted for (e.g. diet or alcohol consumption) in our study is not fully removed with the instrumental variable design. They were however, substantially smaller than in conventional analyses (Suppl. Fig. A). Further, in the instrumental variable analyses, exposure to high or low BMI over the adult life was taken into account, possibly reflecting a genetic disposition to a level of BMI, which may partly explain a stronger associations between BMI and CVD mortality in these analyses.

Less excess deaths among underweight when using the instrumental variable approach than in conventional analyses suggests confounding by midlife factors when using midlife BMI. A stronger association between BMI and CVD mortality in instrumental variable analyses also indicates previous underestimation of the magnitude of the relationship. This is in line with studies using offspring weight as an instrument for own weight in relation to mortality^18,19^. It is also in line with studies based on Mendelian randomization, using genetic variants as instrumental variables for BMI^15–17^. Other outcomes, such as lung disease mortality, may be more strongly influenced by confounding of current illness than CVD. Using instrumental variable analyses on more causes of death in relation to BMI could further shed light on the extent of bias from current illness and other confounders.

A pertinent question has been whether subjects with overweight, in addition to subjects with obesity, are at increased risk of CVD and all-cause mortality. Several studies have reported that obesity appears to increase the risk of CVD, but not overweight^1,2,36^. When applying the instrumental variable approach, however, we observed increased risk of CVD and all-cause mortality also in the overweight range. Importantly, predictions using the instrumental variable approach suggest that the preventive health effect of maintaining a lower population mean BMI is substantially higher than would be predicted using the conventional approach.

## Conclusion

The study suggested a stronger association between BMI and mortality when early life BMI was used as an instrument for midlife BMI, possibly because of less influence of confounding factors, and the inclusion of the aspect of a lifelong exposure.

## Electronic supplementary material

Supplementary Information
