# Supplementary material for: The association between BMI and mortality using early adulthood BMI as an instrumental variable for midlife BMI
Source: Sci Rep. 2018 Jul 31;8:11499. doi: 10.1038/s41598-018-29089-z (PMC6068135; doi:10.1038/s41598-018-29089-z)
Supplement: Supplementary file 1 — Supplementary Information [file 41598_2018_29089_MOESM1_ESM.pdf]

Original research article

The association between BMI and mortality using early adulthood BMI as an instrumental variable for midlife BMI.

Marte K.R. Kjøllesdal[1*], George Davey Smith[2], Inger Ariansen[3], Jonas Minet Kinge[1,3], Eirik Degerud[3], Øyvind Næss[1,3]

1) Institute of Health and Society, University of Oslo, Norway
2) MRC Integrative Epidemiology Unit (IEU), at the University of Bristol, UK
3) Domain for Mental and Physical Health, Norwegian Institute of Public Health, Oslo, Norway

Corresponding author: Marte K.R.Kjøllesdal, E-mail: m.k.kjollesdal@medisin.uio.no. Phone: 0047 22 85 06 78. Institute of Health and society, University of Oslo. Postbox 1130 Blindern, 0318 Oslo, Norway.

Supplementary table A. Associations between Body Mass Index (BMI) measured in midlife and in early adulthood (dependent variable) and risk factors for cardiovascular disease in midlife, in three weight categories. From crude linear regressions.

	BMI, early adulthood (18-20 years) β (95 % CI)	BMI, midlife (40-50 years) β (95 % CI)
Normal- and underweight		
Cholesterol (mmol/L)	-0.02 (-0.02, -0.02)	0.08 (0.08, 0.09)
Systolic Blood Pressure (mmHg)	-0.27 (-0.33, -0.22)	1.48 (1.42 1.54)
Diastolic Blood Pressure (mmHg)	-0.17 (-0.21, -0.14)	0.63 (0.59, 0.67)
Heart Rate (beats/min)	-0.30 (-0.35, -0.25)	-0.98 (-1.03, -0.92)
Smoking, ref: no	1.02 (1.01, 1.03)	0.87 (0.86, 0.87)
Current treatment for hypertension, ref: no	1.10 (0.97, 1.05)	1.17 (1.11, 1.24)
BMI, midlife (kg/m^2)		0.35 (0.34, 0.36)
Overweight		
Cholesterol (mmol/L)	-0.02 (-0.03, -0.02)	0.60 (0.06, 0.08)
Systolic Blood Pressure (mmHg)	-0.25 (-0.30, -0.20)	1.09 (1.01, 1.16)

Diastolic Blood Pressure (mmHg)	-0.15 (-0.18, -0.11)	0.08 (0.80, 0.91)
Heart Rate (beats/min)	-0.17 (-0.21, -0.12)	0.62 (0.54, 0.69)
Smoking, ref: no	1.09 (1.08, 0.10)	0.96 (0.95, 0.97)
Current treatment for hypertension, ref: no	1.01 (0.99, 1.04)	1.26 (1.21, 1.31)
BMI, midlife (kg/m^2)		0.39 (0.38, 0.40)
Obese		
Cholesterol (mmol/L)	-0.01 (-0.02, –0.01)	-0.00 (-0.01, 0.00)
Systolic Blood Pressure (mmHg)	0.05 (-0.03, 0.12)	0.59 (0.50, 0.67)
Diastolic Blood Pressure (mmHg)	0.02 (-0.03, 0.08)	0.34 (0.27, 0.40)
Heart Rate (beats/min)	0.05 (-0.02, 0.12)	0.67 (0.59, 0.75)
Smoking, ref: no	1.09 (1.07, 1.10)	0.98 (0.97, 1.00)
Current treatment for hypertension, ref: no	1.04 (1.02, 1.06)	1.11 (1.09, 1.13)
BMI, midlife (kg/m^2)		0.43 (0.41, 0.44)

Supplementary Table B. Hazard ratios for cardiovascular (CVD) and all-cause mortality with body mass index (BMI) percentiles from cox regressions.

	HR (95 % CI)					
	Model 1	Model 2	Model 3	Model 1	Model 2	Model 3
CVD		Men			Women	
**Midlife**						
10 percentile	1.45 (0.91, 1.43)	1.11 (0.89, 1.39)	1.11 (0.89, 1.40)	0.88 (0.60, 1.30)	0.88 (0.60, 1.30)	0.91 (0.61, 1.34)
10-50 percentile	1	1	1	1	1	1
50-90 percentile	1.12 (0.99, 1.26)	1.07 (0.95, 1.21)	0.93 (0.82, 1.05)	1.22 (0.86, 1.74)	1.14 (0.80, 1.62)	0.91 (0.64, 1.31)
90 percentile	2.43 (2.10, 2.81)	2.19 (1.89, 2.54)	1.47 (1.25, 1.72)	2.20 (1.45, 3.33)	1.91 (0.26, 2.89)	1.20 (0.76, 1.88)
**Early adulthood**						
10 percentile	1.21 (1.00, 1.46)	1.17 (0.97, 1.41)	1.17 (0.96, 1.41)	1.02 (0.62, 1.70)	0.96 (0.58, 1.58)	0.98 (0.59, 1.60)
10-50 percentile	1	1	1	1	1	1
50-90 percentile	1.17 (1.04, 1.32)	1.16 (1.03, 1.31)	1.08 (0.96, 1.22)	1.02 (0.62, 1.70)	1.06 (0.72, 1.40)	0.91 (0.65, 1.26)
90 percentile	2.22 (1.90, 2.58)	2.06 (1.77, 2.40)	1.57 (1.34, 1.84)	1.56 (1.05, 2.32)	1.41 (0.95, 2.09)	1.06 (0.71, 1.59)

All cause						
**Midlife**						
10 percentile	1.53 (1.39, 1.69)	1.50 (1.36, 1.65)	1.38 (0.95, 1.25)	1.24 (1.10, 1.40)	1.25 (1.11, 1.41)	1.24 (1.12, 1.38)
10-50 percentile	1	1	1	1	1	1
50-90 percentile	1.03 (0.97, 1.09)	0.99 (0.94, 1.05)	0.95 (0.90, 1.01)	1.10 (0.97, 1.25)	1.06 (0.94, 1.20)	1.01 (0.89, 1.15)
90 percentile	1.60 (1.47, 1.73)	1.47 (1.35, 1.59)	1.25 (1.14, 1.37)	1.66 (1.42, 1.96)	1.54 (1.31, 1.81)	1.39 (1.18, 1.64)
**Early adulthood**						
10 percentile	1.17 (1.06, 1.28)	1.13 (1.11, 1.48)	1.10 (1.00, 1.21)	1.07 (0.91, 1.26)	1.04 (0.88, 1.22)	1.02 (0.86, 1.20)
10-50 percentile	1	1	1	1	1	1
50-90 percentile	1.12 (1.05, 1.19)	1.11 (1.05, 1.18)	1.05 (1.03, 1.16)	0.97 (0.87, 1.08)	0.96 (0.86, 1.07)	0.93 (0.83, 1.04)
90 percentile	1.57 (1.45, 1.71)	1.48 (1.36, 1.56)	1.32 (1.21, 1.44)	1.30 (1.213, 1.50)	1.24 (1.07, 1.42)	1.12 (0.97, 1.29)

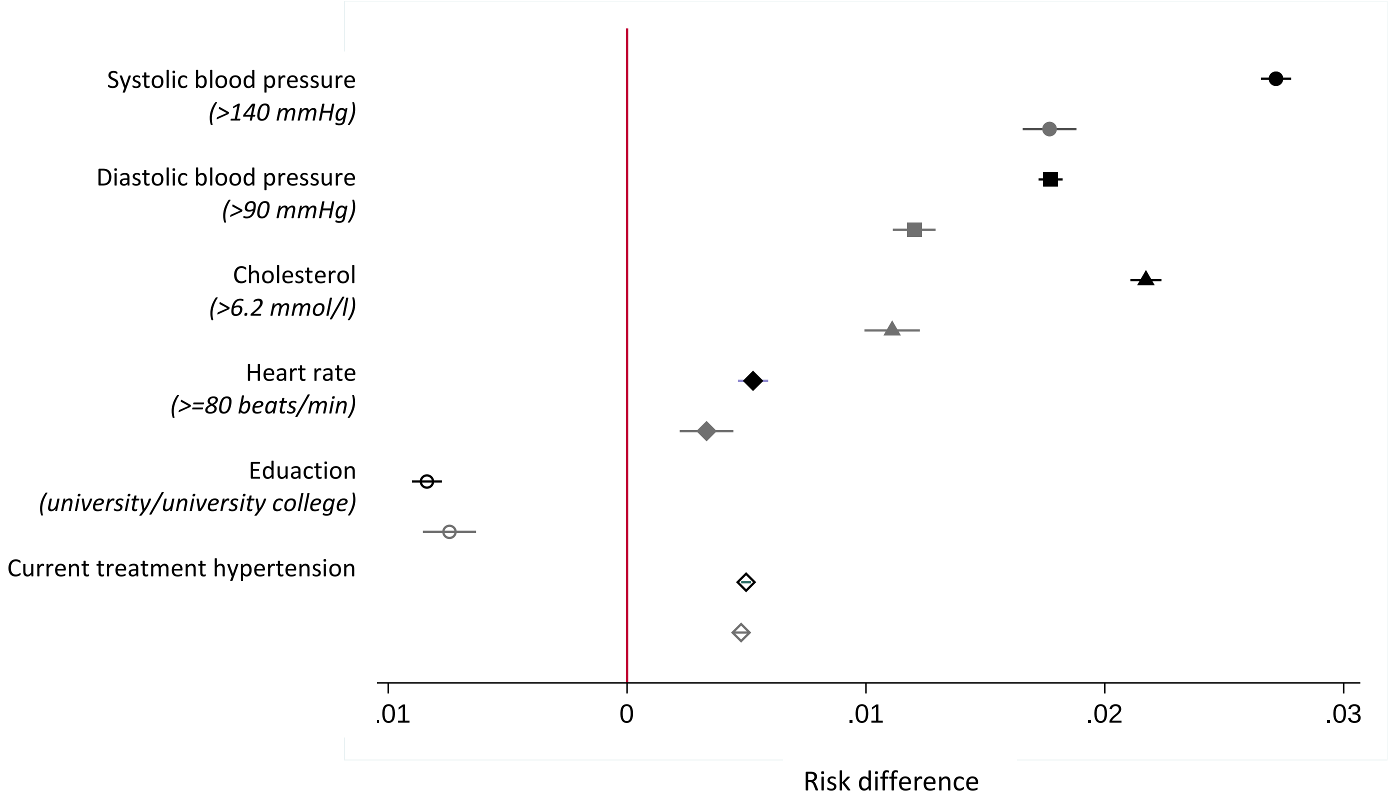

Supplementary Figure A. Covariate balance (with 95 % confidence intervals) by levels of Body Mass Index (black) and instrument (grey).
